# Comparative Study of Peri‐Implant Tissues Following Flapless Placement of a One‐Piece Tissue‐Level Implant Versus Open‐Flap Placement of a Two‐Piece Bone‐Level Implant in a Canine Model

**DOI:** 10.1155/ijod/5542019

**Published:** 2026-06-19

**Authors:** Aurore Barraco, Marie Paule Gustin, Guillaume Noel, Brigitte Grosgogeat, Arnaud Lafon

**Affiliations:** ^1^ Université Claude Bernard Lyon 1, Faculté d’Odontologie, Laboratoire des Multimatériaux et Interfaces (LMI), UMR 5615, Lyon, Cedex 08, France, univ-lyon1.fr; ^2^ Hospices Civils de Lyon, Pôle d’Odontologie, Lyon, France, chu-lyon.fr; ^3^ Université Claude Bernard Lyon 1, Faculté de Pharmacie, Laboratoire de Biométrie et Biologie Évolutive, Équipe Biostatistique Santé, Villeurbanne, UMR CNRS 5558, France, univ-lyon1.fr; ^4^ Hospices Civils de Lyon, Pôle de Santé Publique, Lyon, France, chu-lyon.fr; ^5^ VetAgro Sup, Biovivo/Claude Bourgelat Institut, Marcy-l’Étoile, France, vetagro-sup.fr

**Keywords:** flapless/subcrestal protocol, in vivo, one-piece implant, osseointegration, peri-implant tissues

## Abstract

**Purpose:**

The purpose of this study is to test the hypothesis that the placement of a minimally invasive implant (MII, one‐piece tissue‐level implant) using a flapless/subcrestal protocol provides a better quality of peri‐implant tissues compared to a reference implant (RI, two‐piece bone‐level implant) placed with an open‐flap/crestal protocol in a canine model.

**Methods:**

In this in vivo preclinical study, two types of implants were placed in the mandibula. Clinical, radiological, and histomorphometry measurements were performed at implant placement (T0), at healing abutment placement (T + 8W), and at the end of the study (T + 16W).

**Results:**

Three osseointegration failures occurred, all in the MII group, reducing sample sizes for that group. A significant increase in keratinized tissue height was observed around the MII (3.00 mm ± 1.04 vs 3.68 mm ± 0.63; *p* < 0.001), and a significant decrease was observed for the RI (3.47 mm ± 0.74 vs 2.07 mm ± 0.40; *p* < 0.05) between T0 and T + 16W. The biological width, in mesiodistal sections, was significantly greater around the MII (mean ± standard deviation [SD] peri‐implant mucosa [PM]‐fBIC distances: 3680.5 µm ± 629.2) than for the RI (2065.5 µm ± 395.1, *p* < 0.001). No significant difference was observed in buccolingual sections. Bone remodeling, in mesiodistal sections, around the MII (mean ± SD implant shoulder [IS]‐bone crest [BC] 890.4 µm ± 759.2) was higher around the IS and was significantly greater around the RI (mean ± SD IS‐BC −283.4 µm ± 285.3, *p* < 0.001), for which significant bone resorption was observed.

**Conclusion:**

In this canine model and under the specific study conditions, the MII was associated with distinct patterns of bone remodeling and greater epithelial–connective tissue dimensions in mesiodistal sections. These results suggest a favorable soft tissue environment around the MII. It would be interesting to confirm these results in humans, particularly the elderly and medically compromised patients for whom the atraumatic flapless surgical protocol seems particularly suitable.

## 1. Introduction

Just as keyhole surgery has transformed medical procedures, the development of minimally invasive techniques in dental implantology will enable better adaptation to the needs of an increasingly elderly and medically compromised population [1–3]. Indeed, minimally invasive techniques decrease the risk of pre‐ or postoperative complications such as infections or hemorrhages and shorten the healing period [4].

The flapless technique enhances patient postsurgical comfort through the use of an atraumatic surgical act and reduces the number of surgical procedures, such as bone grafting and the need to remove sutures or place healing abutments [5, 6]. These therefore help to reduce the cost and healing time of the treatment [4, 7–9].

Several parameters are essential to ensure the long‐term survival of implants, especially in elderly, medically compromised, or handicapped patients, such as the quality and quantity of the keratinized tissue around the implant [10, 11]. Similarly, for the long‐term success of implants, the integrity of the biological width and the thickness of the soft tissues are now considered crucial parameters in order to avoid bacterial infiltration and bone loss [10–13]. These parameters might also be influenced by the surface treatment and the implant design [14–16]. In flapless surgery, another important parameter for long‐term success is the level of implant embedding, in particular that of the machined smooth neck and the rough surface of one‐piece implants. For instance, Hermann et al. [17] have shown that a subcrestal placement of the machined smooth neck results in more crestal bone loss than an equicrestal placement and that a supracrestal placement can lead to bone apposition; however, subcrestal placement increases the vertical soft tissue thickness that creates a stronger barrier to bacterial infiltration by increasing the amount of keratinized tissue [18]. One limitation of flapless surgery is the need for precise implant positioning, especially the exact level of bone embedding that is difficult to control through the lack of visibility of the bone crest (BC). For this reason, a new implant with a design adapted to flapless surgery was developed in 2008. This minimally invasive implant (MII, one‐piece tissue‐level implant) uses a specific flapless/subcrestal protocol for predictable results that respect the peri‐implant tissues.

The purpose of the present in vivo study is to test the hypothesis that the placement of an MII using a flapless technique provides a better quality of peri‐implant tissues compared to a reference implant (RI, two‐piece bone‐level implant) that uses an open‐flap/crestal protocol.

## 2. Materials and Methods

### 2.1. Ethics Statement

This in vivo preclinical study was conducted in compliance with EU Directive 2010/63/EU for animal experiments and with ARRIVE guidelines [19]. The protocol of this study was submitted to the ethics committee of VetAgro Sup and authorized by the French Ministry of Higher Education and Research under Project Number APAFIS#6103‐2016070511504068.

### 2.2. Animals

**Figure 1:**
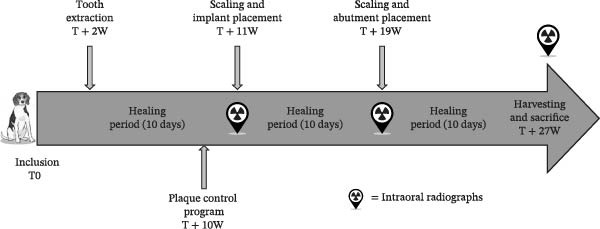
Experimental protocol (T: time; W: weeks).

Five male beagle dogs (12 months old and weighing ~10 kg) with fully erupted permanent dentition were included. They were of the Marshall US strain, immunocompetent, not genetically modified, selected by the breeder, and from the French site of Marshall BioResources located in Gannat (Allier department, French administrative area). During all experiments, the dogs were housed in cages maintained at 19 ± 2°C and 35%–70% humidity, with a 12/12h light/dark cycle. They were fed with a soft diet (SAFE 326; Safe Lab, Rosenberg, Germany) and water ad libitum. The experimental part of the study was started after an adaptation period of 2 weeks. Control visits and tooth brushing were performed twice a week. The experimental protocol of the study is shown in Figure 1.

### 2.3. Implant Devices

**Figure 2:**
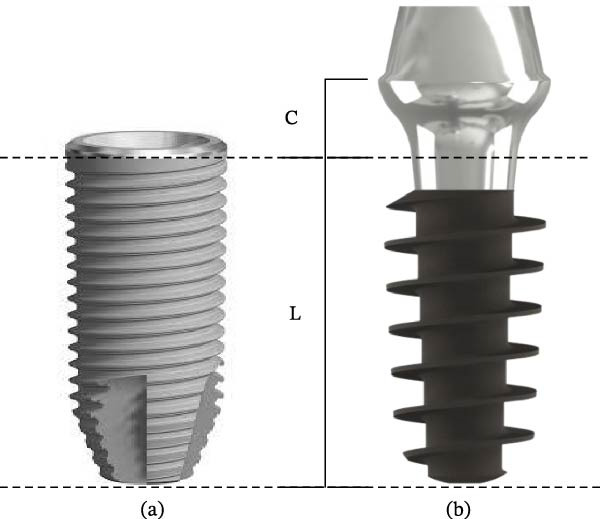
(a) Reference implant (RI) Nobel Parallel CC implant: bone‐level implant with conventional triangular threads and the implant length including 7 mm of rough surface and (b) minimally invasive implant (MII) MagiCore implant: tissue‐level implant with fin rectangular threads (Magic Fin Thread), a machined smooth neck (Cuff), a prosthetic platform (post) and the implant length including 6 mm of rough surface and 1 mm of machined surface (L).

Two different types of implants were used in this study: an MII (MagiCore, InnoBioSurg implant, Daejon, Republic of Korea) and an RI (Nobel Parallel CC Nobel Biocare, Kloten, Switzerland). The MII was 4 mm in diameter and 7 mm in length, with 6 mm of rough surface and 1 mm of machined surface. This one‐piece titanium grade 5 sandblasted implant can be divided into three parts: a tapered body with 0.15 mm‐thick rectangular threads (Magic Fin Thread); a machined smooth neck (Cuff) with four different available heights chosen according to the gingival height that allows connective tissue and junctional epithelium adhesion; and a prosthetic platform to support the future prosthetic restoration (post; Figure 2b). The RI was 3.75 mm in diameter and 7 mm in length. This two‐piece titanium grade 4 anodized (TiUnite) implant has a conventional configuration (tapered, self‐tapping, and triangular threads; Figure 2a).

### 2.4. Surgical Protocol

**Figure 3:**
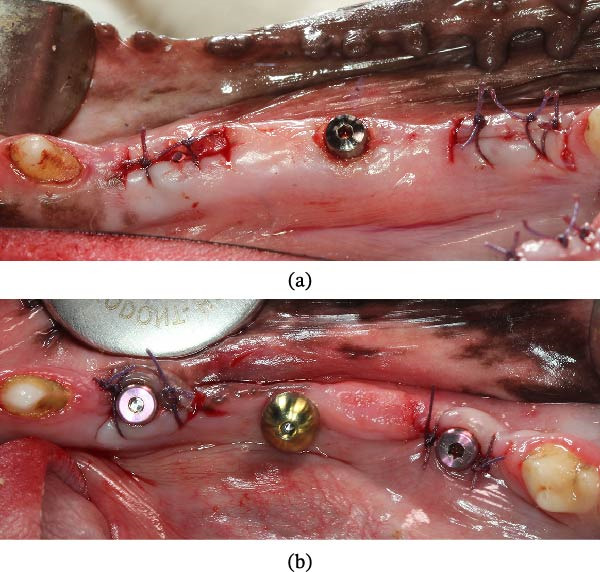
(a) Implant placement (T0) and (b) healing abutment placement (T + 8W) with reference implant (RI) on sites 1 and 3, and MII on site 2.

All procedures were performed by an experienced surgeon under sterile conditions and general anesthesia; the latter was induced using intravenous injection of ketamine (3 mg/kg) and medetomidine (20 µg/kg) and maintained using isoflurane inhalation. For each surgery, the dogs received an injection of amoxicillin once a day from the day before the surgery and for 7 days to prevent any infection. The dogs also received oral cavity disinfection with chlorhexidine 0.2% and local anesthesia using articaine and 1:100,000 epinephrine. For pain control, the animals received three intravenous injections of morphine: for premedication, at the end of the procedure, and 4 h after extubation (0.2 mg/kg). A transcutaneous fentanyl patch was placed on the day of surgery (75 µg/h). Meloxicam was given to the animals for 10 days (0.2 mg/kg/day the first day and 0.1 mg/kg/day the next 9 days). During the first surgery, the mandibular second, third, and fourth premolars, along with the first molar, were carefully extracted on both sides. After an 8‐week healing period, a total of 30 implants (15 MII and 15 RI) were allocated in the mandible of each dog, by site within each dog, alternating MII and RI positions (left/right) across the three sites to balance tooth position (Figure 3a). However, the impossibility of taking a 3D intraoperative image, as recommended by the manufacturer, made it difficult to verify the correct bone embedding of the MII. The design of the MII includes a 1 mm machined smooth surface along its length, which is embedded during flapless surgery according to the manufacturer’s protocol. For the RI placed at the level of the crestal bone, a conventional two‐stage surgical protocol with a mucoperiosteal flap elevation and sequential drills for implant osteotomy was performed following the manufacturer’s instructions (Figure 3b). 8 weeks after implant placement, healing abutments were connected to each implant with a small mid‐crestal incision for the RI. For MII, flapless surgery was conducted. At T0, the placement protocol involves a single surgical step without a flap, at the end of which a cover screw was placed (Figure 3a). At T8, a healing cap was placed without tissue management.

### 2.5. Clinical Analysis

The height of the keratinized tissue (mm) around the implant was clinically evaluated at six different probing points and also on histological sections after the animals were sacrificed at the end of the study. Implant stability for the RI and the MII was measured using Periotest (Dentisystem, Bese, Hungary) at implant placement (T0), healing abutment placement (T + 8W), and at the end of the study (T + 16W). The Periotest scale extends from −8 to + 50 Periotest value (PTV); the lower the value, the greater the stability of the measured implant [20]. Before harvesting at T + 16W, peri‐implant inflammation was measured using a periodontal probe scored using Carter and Barnes’s [21] gingival bleeding index (0 = no bleeding, 1 = bleeding). Plaque index was measured using Loe [22] and Silness’s plaque index (0 = no plaque in the gingival area; 1 = presence of a film of plaque that may only be recognized by running a probe across the tooth surface; 2 = moderate accumulation of soft deposits, which can be seen by the naked eye; 3 = abundance of plaque).

### 2.6. Radiological Analysis

Intraoral radiographs were taken at implant placement (T0), at healing abutment placement (T + 8W), and at the end of the study (T + 16W). Angulators were customized (Unifast TRAD resin; GC, Lucerne, Switzerland) to ensure precise placement of receptors, and radiographs were standardized to minimize distortion and angulation errors. An image analysis program (ImageJ v1.46h, National Institutes of Health, Bethesda, MD, US) was used to superimpose the radiographs taken at implant placement (T0) and the end of the study (T + 16W) to measure the peri‐implant marginal remodeling. This peri‐implant marginal remodeling was calculated by the difference (mm) between the distance from the apex to the fBIC at the end of the study (T + 16W) compared to the implant placement (T0).

### 2.7. Sample Collection and Histologic Preparation

The animals were sacrificed 4 months after implant placement with an intravenous lethal dose of pentobarbital. The samples were then collected using a trephine under profuse saline irrigation and fixed with a mixture of 2% glutaraldehyde and 2% paraformaldehyde in sodium cacodylate buffer for 1 week. Dehydration was performed in serial steps of increasing ethanol concentration, and the blocks were finally embedded in methyl methacrylate. Nondecalcified sections were prepared using a method adapted from Donath and Breuner [23]. Two cuts were performed for the polymerized blocks: one mesiodistal and one buccolingual, parallel to the long axis of each implant using a diamond wire saw. Then, the sections were obtained and reduced by microgrinding and polishing to a thickness of about 40 µm. These sections were then superficially stained with a modified paragon stain and digitized for histomorphometric quantification.

### 2.8. Histomorphometric Analysis

**Figure 4:**
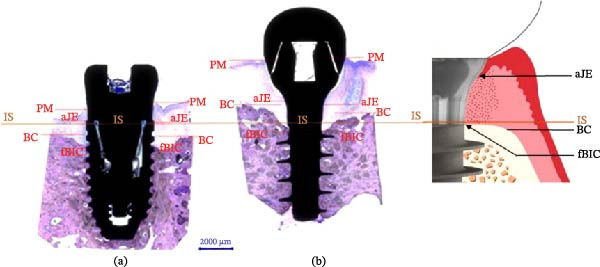
Mesiodistal section of (a) RI and (b) MII with the following landmarks: PM, the marginal position of the peri‐implant mucosa; aJE, the apical termination of the pocket epithelium; fBIC, the most coronal bone‐to‐implant contact; BC, the top of the bone crest; and IS, the implant shoulder.

The images were analyzed by two different investigators using ImageJ (v1.46h, National Institutes of Health). Before the analysis, the two investigators used randomly selected sessions from other animal studies for calibration. The following measurements were performed for each section, and a mean was calculated on the left and right sides for the mesiodistal and buccolingual sections. The following landmarks were identified for linear measurements (µm): the marginal position of the peri‐implant mucosa (PM); the apical termination of the pocket epithelium (aJE); the most coronal bone‐to‐implant contact (fBIC); the top of the BC; and the implant shoulder (IS; Figure 4).

The primary endpoint was the height of the biological width (PM‐fBIC). Secondary endpoints were bone resorption/apposition (IS‐BC), a negative value (−) was given when BC was below IS [24], height of epithelial tissue (PM‐aJE), height of connective tissue (aJE‐fBIC), the peri‐implant crestal bone level (IS‐fBIC), and marginal bone level (BC‐fBIC). The percentage of bone‐to‐implant contact (%BIC) was also calculated for each mesiodistal section; this corresponded to the percentage of the height of the bone in direct contact with the implant and the bone and the length of the total surface of the implant [25].

All measurements were performed on coded sections, with the examiner blinded to the implant type.

### 2.9. Statistical Analysis

To compare the primary endpoint (PM‐fBIC) between the two implants with a large effect size d of 0.8 according to Cohen [26], a sample of 15 values per implant was considered sufficient to ensure 80% power to detect an effect size of 0.80; a total of five dogs, each with six implants, were therefore used in the present study.

To evaluate the interexaminer reliability between the two investigators for the histomorphometric and radiographic analyses, the Lin’s [27] concordance correlation coefficient (CCC) for agreement on a continuous measure was calculated (function epi.ccc of R package Epi) [28]. Only the global CCC that measures the overall concordance (precision and accuracy) was reported; a perfect concordance occurs when CCC = 1. In case of good agreement, the mean of both values was retained in further analyses.

Quantitative and ordinal data were reported as mean with standard deviation (SD) and qualitative binary data as frequency with relative frequency, *n* (%). To compare data between implants, a linear mixed effects model was fitted with implant, site, and side as an explanatory variables and a random intercept for dogs. We reported the *p*‐value of the site‐side adjusted model. We fitted a model with family Gaussian and link identity for quantitative variables (function lme, R package nlme), with family binomial and logit link for binary variables (function glmer, R package lme4), and a cumulative logit link model for ordinal variables (function clmm, R package ordinal). The effect of the implant was stratified by time in the mixed model only for the primary stability (PTV). Assumptions of the models were checked.

For all tests, a *p*‐value less than 0.05 was considered significant. In this exploratory study, all variables were interpreted independently, and there was no need to control the family‐wise error rate. Then, no adjustment was made for the multiple comparisons. Statistical analyses were performed using the R language Version 4.1.0, available at http://cran.r-project.org.

## 3. Results

### 3.1. Clinical Findings

All the animals survived, and all the extracted teeth healed well before the implant placement. Three of the 15 implants were not osseointegrated; all three were MII. No missing data imputation was performed.

**Figure 5:**
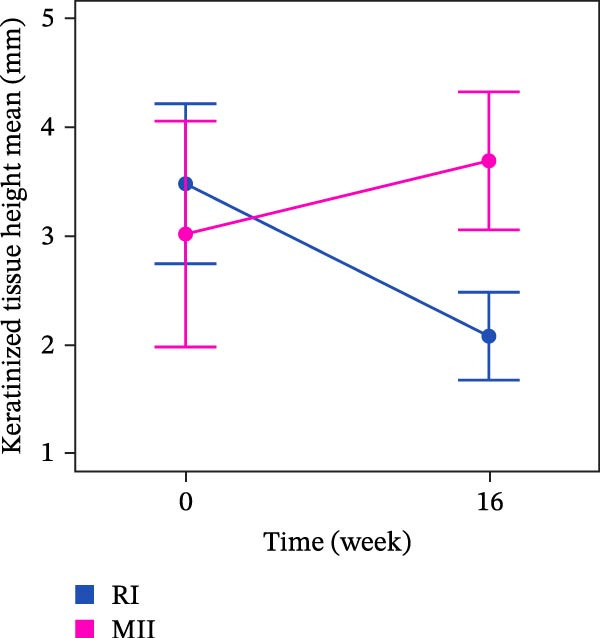
Height of keratinized tissue between RI and MII groups at inclusion and T + 16W. The circles and the vertical bars correspond to the mean and the standard deviation, respectively.

The mean ± SD height of keratinized tissue around the MII significantly increased between implant placement (T0; 3.00 mm ± 1.04) and the end of the study (T + 16W; 3.68 mm ± 0.63; *p* < 0.001), and significantly decreased around the RI (3.47 mm ± 0.74 vs. 2.07 mm ± 0.40; *p* < 0.05; Table 1, Figure 5). There was no significant difference regarding the bleeding and plaque indexes at the end of the study (T + 16W; Table 1).

**Table 1:** Comparison of clinical parameters between RI and MII groups at implant placement (T0) and at the end of the study (T + 16W).

Keratinized tissue height^a^ (mm)	*n*	T0	*n*	T + 16W
RI	15	3.47 ± 0.74	15	2.07 ± 0.40 ^∗^
MII	12	3.00 ± 1.04	11	3.68 ± 0.63^‡^
Gingival bleeding index, *n* (%)				
RI	15	NA	—	5 (33.3)
MII	12	NA	—	4 (33.3)
Plaque index (%)				
RI	15	NA	—	1.27 ± 0.83
MII	12	NA	—	1.58 ± 1.16

*Note:* Quantitative variables: mean ± SD; binary variable: *n* (%). T0 vs. T + 16W:  ^∗^ *p* < 0.05; ^‡^ *p* < 0.001 using two independent site‐side adjusted models for each time for keratinized tissue height variable.

Abbreviations: NA, not applicable; T, time; W, weeks.

^a^Value measured clinically at T0 and histologically at T + 16W.

Both groups had excellent stability (from −8 to 0 PTV) with no significant difference between the MII and the RI at implant placement (T0) and the end of the study (T + 16W), but there was a significantly greater stability in the RI group at healing abutment placement (T + 8W; mean PTV: 4.14 ± 1.73 vs −1.83 ± 3.01, *p* < 0.01; Table 2).

**Table 2:** Comparison of primary stability parameters between RI and MII groups at the different follow‐up times.

Primary stability (PTV)	Time period					
	*n*	T0	*n*	T + 8W	*n*	T + 16W
RI	15	−3.80 ± 1.56	15	−4.14 ± 1.73	15	−3.12 ± 1.22
MII	15	−3.38 ± 1.97	12	−1.83 ± 3.01^a^	12	−2.11 ± 2.59

*Note:* Comparison of RI and MII groups at each follow‐up time.

Abbreviations: PTV, Periotest value; T, time; W, weeks.

^a^ *p* < 0.01, using site‐side an adjusted mixed‐effects model stratified on time.

### 3.2. Radiological Findings

There was high agreement between the two examiners with CCC > 0.997. Peri‐implant bone remodeling (apex‐*f*BIC) between implant placement (T0) and the end of the study (T + 16W) was significantly higher around the MII (mean ± SD bone level difference 2.58 mm ± 1.97) than around the RI (0.63 mm ± 0.36, *p* < 0.001; Table 3).

**Table 3:** Results from radiological measurements of the peri‐implant bone remodeling (apex‐fBIC) at the implant placement (T0) versus at the end of the study (T + 16W).

	RI	MII
Bone remodeling (apex‐fBIC) (mm)	0.63 ± 0.36	2.58 ± 1.97^a^

*Note:* Mean ± SD (mm).

^a^ *p* < 0.001 using a site‐side adjusted mixed‐effects model.

### 3.3. Histological Findings

There was high agreement between the two examiners with CCC > 0.993.

#### 3.3.1. Soft Tissue Behavior

**Figure 6:**
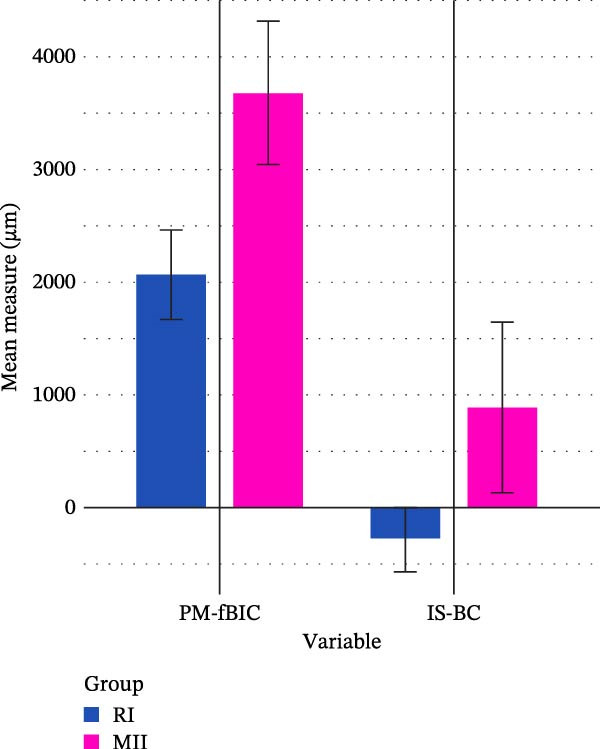
Comparison of histological parameters between RI and MII groups at T + 16W for biological width (PM‐fBIC) and marginal bone level (IS‐BC) in mesiodistal sections. Error bars are standard deviation.

For soft tissue mesiodistal sections, the mean ± SD PM‐fBIC (3680.5 µm ± 629.2 vs. 2065.5 µm ± 395.1, *p* < 0.001) and PM‐aJE distances (2327.1 µm ± 322.9 vs. 889.9 µm ± 435.1, *p* < 0.001) were significantly greater for the MII than for the RI. There was no significant difference in aJE‐fBIC between the two groups. For buccolingual sections, there was no significant difference between the two groups in terms of PM‐fBIC, PM‐aJE, and aJE‐fBIC (Table 4 and Figure 6).

**Table 4:** Histomorphometric findings at the end of the study (T + 16W).

	*n*	RI	*n*	MII
Mesiodistal section				
PM‐fBIC (µm)	15	2065.5 ± 395.1	11	3680.5 ± 629.2^‡^
IS‐BC (µm)	15	−283.4 ± 285.3	11	890.4 ± 759.2^‡^
PM‐aJE (µm)	15	889.9 ± 435.1	11	2327.1 ± 322.9^‡^
aJE‐fBIC (µm)	15	1175.8 ± 358.3	11	1353.3 ± 513.3 (*p* = 0.43)
IS‐fBIC (µm)	15	655.2 ± 276.5	11	802.9 ± 646.2 (*p* = 0.48)
BC‐fBIC (µm)	15	400.3 ± 162.1	11	1692.3 ± 671.4^‡^
BIC (%)	15	0.695 ± 0.147	11	0.517 ± 0.251 ^∗^
Buccolingual section				
PM‐fBIC (µm)	13	3064.0 ± 948.8	10	2889.6 ± 989.7 (*p* = 0.78)
IS‐BC (µm)	15	1424.9 ± 1039.0	12	253.6 ± 1200.5^†^
PM‐aJE (µm)	12	1516.7 ± 631.8	10	1532.0 ± 775.8 (*p* = 0.78)
aJE‐fBIC (µm)	13	1611.4 ± 949.8	10	1357.9 ± 546.6 (*p* = 0.50)
IS‐fBIC (µm)	15	1466.8 ± 1056.2	12	940.6 ± 1070.4 (*p* = 0.21)
BC‐fBIC(µm)	15	41.8 ± 170	12	687 ± 506.3^‡^

*Note: n*, number of samples analyzed; mean ± standard deviation (µm) and comparison of MII and RI groups  ^∗^ *p* < 0.05; ^†^ *p* < 0.01; ^‡^ *p* < 0.001 using site‐side adjusted mixed‐effects models; negative values may be observed for IS/BC distance as the BC point may be located below the IS point. Epithelial tissue (PM‐aJE), connective tissue (aJE‐fBIC), biological width (PM‐fBIC), peri‐implant crestal and marginal bone levels (BC‐fBIC, IS‐fBIC, and IS‐BC), and the bone‐implant contact (%BIC).

#### 3.3.2. Hard Tissue Behavior

For bone remodeling in mesiodistal sections, the mean ± SD IS‐BC distance was negative for the RI (−283.4 µm ± 285.3) and positive for the MII (890.4 µm ± 759.2), and there was a significant difference between these values (*p* < 0.001). The mean ± SD BC‐fBIC was significantly greater for the MII (1692.3 µm ± 671.4) than for the RI (400.3 µm ± 162.1, *p* < 0.001). For bone remodeling in buccolingual sections, the mean ± SD IS‐BC distance was significantly greater for the RI (1424.9 µm ± 1039.0) than for the MII (253.6 µm± 1200.5, *p* < 0.01; Table 4 and Figure 6). There was a trend towards a greater mean ± SD IS‐fBIC distance for the RI (1466.8 µm ± 1056.2) than for the MII (940.6 µm ± 1070.4, *p* = 0.20), and the mean ± SD BC‐fBIC distance was significantly greater for the MII (687 µm ± 506.3) than for the RI (41.8 µm ± 170, *p* < 0.001; Table 4).

The mean %BIC was significantly greater for the RI (0.695 µm ± 0.147) than for the MII (0.517 µm ± 0.251, *p* < 0.05; Table 4).

## 4. Discussion

The objective of this in vivo preclinical study was to analyze the quality of peri‐implant tissues around a RI + open‐flap/crestal protocol compared to MII + flapless/subcrestal protocol. The results indicate that hard and soft tissue behavior differed between the MII and the RI. These differences may be explained by the design of the implants tested, in particular the tissue behavior around the machined smooth neck of the MII.

Regarding hard tissue behavior, it is now well established that the absence of thread exposure, peri‐implant tissue thickness, and position of the microgap between the collar and the prosthesis improve the stability of peri‐implant tissues [29]. Bone quality is also pivotal; Simons et al. [30] reported that early crestal bone loss around the neck of mandibular implants was greater when the bone was cortical than when it was cancellous. For bone‐level implants, platform switching and the platform‐neck design reduce the risk of exposure of rough threads and the occurrence of peri‐implantitis [16, 31, 32]. Regarding one‐piece implants, such as the MII, the absence of a microgap limits the risk of bone resorption. In the present study, no significant findings were observed in the buccolingual sections, which may be attributed to the study’s limitations, particularly the lack of 3D imaging. Conversely, in the mesiodistal sections, we observed for the IS‐BC a negative mean value for the RI and a positive mean value for the MII. No significant difference was found when considering only the IS‐fBIC distance, but the results seemed to show lower bone resorption in buccolingual sections around the MII. The RI was placed at the BC level, and the machined smooth neck of the MII was placed subcrestally. However, at T + 16W, an apical displacement of the crestal bone was observed around the RI compared to MII (IS‐BC). It was also observed that BC was higher and the biological width was longer around MII, as confirmed by significantly higher mean values of PM‐aJE and PM‐fBIC on mesiodistal sections. The greater PM‐fBIC distance in the MII group reflects both a coronal positioning of the soft tissue margin and an apical repositioning of the BC; whether the net biological outcome is favorable requires longer‐term evaluation, including assessment of crestal bone stability beyond 16 weeks. It is of note that several authors have reported that there is no significant difference in osseointegration and peri‐implant bone remodeling between two‐stage and one‐stage (with flap) surgical protocols with similar placement depth [33–35]. The observed differences in the present study could be explained by the use of a flapless technique for the MII; however, intraoperative 3D images that are part of the protocol were not possible in this animal study. Consequently, the level of bone embedding of the rough surface of the MII implant could only be assessed using 2D imaging. As a result, we cannot exclude the possibility that some threads were positioned outside the bone in the buccolingual dimension, particularly in areas with narrow bone ridges. In addition, the oral intubation of the dogs made it impossible to check the existence of any occlusal contacts, resulting in occlusal contact between the antagonist teeth and the MII. The differences in crestal bone remodeling between the MII and the RI are in accordance with those reported by Hermann et al., [17] who found significant cortical bone resorption on a smooth neck when comparing bone‐level and tissue‐level implants [36–38]. The differences also align with a meta‐analysis that found that the marginal bone loss around rough threaded neck implants was significantly less than around the machined smooth neck in subcrestal placement [39]. This was confirmed by the radiological findings herein, showing that the peri‐implant bone remodeling (apex‐fBIC) between implant placement (T0) and the end of the study (T + 16W) was significantly greater around the MII than around the RI.

Regarding soft tissue behavior, as described in the literature, only gingival cells can adhere to the smooth neck, and the bone initially present around the collar was replaced by soft tissues [40, 41], as found herein. The resultant larger epithelio‐conjunctival attachment for the MII could create a barrier to bacterial infiltration. Furthermore, the clinical results show a reduced height of the keratinized tissue at implant placement (T0) around the MII, which can be explained by the removal of the keratinized tissue in the MII surgical protocol (drill trephine). But after tissue maturation at T + 16W, the results were inversed; the keratinized tissue was significantly thicker around the MII. This observation was confirmed by the histological results that showed a higher mean soft tissue height around the MII between implant placement (T0) and the end of the study (T + 16W), whereas there was a significant mean soft tissue reduction around the RI. This phenomenon may indicate that a minimally invasive procedure along with a one‐piece smooth neck implant develops a sufficiently dense epithelial barrier and thick keratinized soft gingival tissue to prevent peri‐implantitis [42]. This hypothesis is supported by the results of an in vitro study by Attik et al. [16] showing that adhesion and proliferation of human gingival fibroblasts are higher on the MII than on the RI under the scanning electron microscopy. Several studies have demonstrated the benefits of smooth surfaces at the neck of RI implants, which allow for the attachment of gingival soft tissues [42, 43] to create a protective barrier over the underlying bone and improve long‐term soft tissue stability [44].

Finally, the thicker epithelial–connective tissue barrier observed around the MII in this model, only in mesiodistal and not buccolingual sections due to the described limitations of this study, may contribute to a more robust soft tissue seal, which is hypothesized to reduce bacterial infiltration; however, this study was not intended to investigate peri‐implantitis. These arguments explain the appeal of MII implants. They help us understand the tissue behavior of a smooth neck positioned subcrestally compared to an RI positioned at the bone level. Indeed, embedding a smooth neck allows the rough part of the implant to be positioned deeper in the cancellous bone, thereby anticipating resorption of the thinner and more fragile cortical bone at the neck of the implant and promoting better quality peri‐implant tissues.

For %BIC, the differences in surface treatment and thread design of the RI and the MII can explain the observed differences, as reported by Abrahamsson and Berglundh [43], who found that microthread implants had a larger %BIC in comparison to macrothread implants.

In addition, the absence of a significant difference in the mean gingival bleeding index between the two groups suggests that neither implant is more favorable for the development of peri‐implant inflammation. The greater frequency of plaque index 3 on the MII can be explained by the wide MII platform design compared to the RI, which could be of importance as it is recognized that the presence of plaque leads to more bone resorption [44, 45].

Concerning implant stability, the significant increase observed at healing abutment placement (T + 8W) for the RI can be explained by the difference in thread design and the implantation protocol; the RI, self‐tapping implants, obtains stability through undersized drilling and cortical bone compression, in contrast with the adjusted implantation without bone compression (Magic Fin Thread) through fine morphological contact in the cancellous bone of the MII. Furthermore, a study by Coutant et al. [46] showed that the primary stability of the Magic Fin Thread was compatible with clinical osseointegration in sinus sites with severe bone atrophy (posterior maxillary areas with type IV bone and residual bone height 4 mm) [47].

## 5. Limitations

The aim of this in vivo preclinical study was to compare two different surgical protocols (with and without the opening of a flap), and these protocols differed in three aspects (submerged versus transgingival healing, flap versus no flap, and different implant designs), which could be considered a limitation. We deliberately did not confine ourselves to comparing two different implant designs (bone level vs. tissue level) or different levels of implant embedding; rather, we investigated the advantages of placing an MII in terms of tissue quality compared to an RI placed using conventional flap surgery. The comparison of identical mucosal healing between the two implants would only be possible if the RI implants and abutment were placed at the same time. We chose to follow the standard protocol for placing a BL implant with two surgical steps. This procedure is used clinically, where RI implants can be put into function from 8 weeks, according to the available data. The placement of a healing abutment on the RI at the time of implant placement would have altered the hypothesis of comparing surgical protocols. This decision can indeed be considered a limitation for soft tissue healing. Another limitation is the absence of 3D imaging and intubation through the mouth of the dogs, which could have influenced the results; however, unlike this animal study, clinicians who use MII will be able to verify the implant depths. Furthermore, the canine model does not allow an assessment of infectious and hemorrhagic risk factors, and therefore, it is difficult to generalize the results to medically compromised patients. A clinical study targeting this population, respecting the MII insertion protocol and using 3D imaging, could be conducted to verify the results of the present study.

## 6. Conclusion

In this canine model and under the study conditions, the MII + flapless/subcrestal protocol was associated with larger epithelial–connective tissue dimensions in mesiodistal sections and distinct patterns of crestal bone remodeling compared to the RI + open‐flap/crestal protocol. However, the discussed limitations of this study prevent attributing any observed difference to a single factor. These findings support further investigation in a controlled human clinical study, particularly in elderly and medically compromised patients.

**Nomenclature**
aJE: Apical junction epithelial
APAFIS: Authorization Number for Projects Using Animals for Scientific Purposes
ARRIVE guidelines: Animal Research: Reporting of In Vivo Experiments
C.b: Bias correction factor
BC: Bone crest
BL: Bone‐level implant
EU: European Union
fBIC: First bone to implant contact
IS: Implant shoulder
MII: Minimally invasive implant
PM: Peri‐implant mucosa
RI: Reference implant
SD: Standard deviation.

## Acknowledgments

The authors would like to thank Philip Robinson (DRS, Hospices Civils de Lyon) and Julia Mwenge Wambel for manuscript preparation.

## Funding

This study was supported by an industriel partnership with InnoBioSurg Co., Ltd. (Daejeon, Republic of Korea). InnoBioSurg provided the devices and partial financial support.

## Disclosure

A preprint has previously been published [48]. InnoBioSurg had no role in data collection, analysis, interpretation, or the decision to publish. The study was conducted and analyzed independently at the Laboratoire des Multimatériaux et Interfaces (UMR CNRS 5615).

## Ethics Statement

All applicable international guidelines for the care and use of animals were complied according to the Institutional Animal Care Guidelines of the French National Ministry of Research.

## Conflicts of Interest

The authors declare no conflicts of interest.

## Data Availability

The data that support the findings of this study are available upon request from the corresponding author. The data are not publicly available due to privacy or ethical restrictions.
